# Understanding the Role of Patient-Reported Outcomes for Decision-Making in Early-Phase Dose-Finding Clinical Trials

**DOI:** 10.3390/curroncol32030176

**Published:** 2025-03-19

**Authors:** Richard Brown, Nolan A. Wages, Li Liu, Arnethea L. Sutton, Andrew S. Poklepovic

**Affiliations:** 1 Department of Social and Behavioral Sciences, School of Public Health, Virginia Commonwealth University, Richmond, VA 23284, USA; 2 Department of Biostatistics, School of Public Health, Virginia Commonwealth University, Richmond, VA 23284, USA; 3 Department of Kinesiology and Health Sciences, College of Humanities and Sciences, Virginia Commonwealth University, Richmond, VA 23284, USA; 4 Department of Internal Medicine, School of Medicine, Virginia Commonwealth University, Richmond, VA 23284, USA

**Keywords:** early phase, dose finding, patient-reported outcomes, patient–physician communication, community outreach and engagement

## Abstract

In early-phase dose-finding clinical trials, integrating patient-reported outcomes (PROs) is essential for enhancing patient-centered decision-making. This short communication advocates for several key practices to achieve such integration. Firstly, foster patient-centered communication that ensures patient understanding of the potential benefits of early-phase trials, thereby mitigating therapeutic misconceptions. Secondly, (a) facilitate partnerships to understand and address the underlying reasons for discrepancies between clinician and patient reports of adverse events and (b) facilitate partnerships among clinical trialists, statisticians, clinicians, patients, and advocates to gain diverse perspectives of adverse events and in so doing ensure that patients comprehend how their data will be used. Thirdly, optimize trial design and data collection by (a) determining optimal and feasible frequencies for PRO collection to minimize patient burden while maintaining data integrity and (b) effectively incorporating concordant PROs to guide dose recommendation decisions and adapt trial designs and statistical methods accordingly. Future research will involve investigating the application of these practices in patients within the Virginia Commonwealth University (VCU) Massey Comprehensive Cancer Center Catchment Area. By integrating these recommendations, early-phase dose-finding clinical trials have the potential to achieve more informed and patient-centered objectives.

## 1. Introduction

Early-phase dose-finding trials are essential for the development of novel oncology treatments [1]. These trials are designed to test the toxicity of new agents in human subjects to determine the acceptable dose levels for subsequent studies, to identify how a cancer agent should be given, or to observe how an agent affects the body [2]. These trials are generally conducted in late-stage cancers when other treatment modalities have failed. Traditionally, Phase I trials have focused primarily on physician-reported toxicity assessments, relying on adverse events (AEs) captured using the National Cancer Institute’s Common Terminology Criteria for Adverse Events (NCI-CTCAE). There are now myriad modalities for treating cancer, ranging from immunotherapy, cellular therapy, targeted therapy, to cytotoxic therapy. The time course and intensity of the toxicities for these therapies can vary widely, ranging from acute high-grade toxicity in the case of CAR-T or adoptive cell therapy to chronic and late toxicity from prolonged exposure to targeted therapy and immunotherapy. The emergence of advanced, targeted cancer therapies, novel immunotherapies alongside initiatives like the U.S. Food and Drug Administration’s (FDA) Project Optimus, underscores the need to reshape dose-finding practices to better incorporate the patient’s voice. Project Optimus aims to reform the dose optimization and selection paradigm in oncology drug development by shifting the focus from merely identifying the maximum tolerated dose (MTD) to determining the optimal biological dose (OBD) that balances efficacy and safety, thereby improving patient outcomes [3]. Patient-reported outcomes (PROs) have shown potential to effectively capture subjective AEs, yet they are currently included in only a minority of dose-finding trials, with even fewer using them to make real-time dose adjustments. Specific PROs designed around the expected toxicity profile will be valuable. Additionally, toxicities that have major impacts on QOL may not be adequately captured in the CTCAE grading system or remain undervalued in their impacts. For example, musculoskeletal toxicity/arthralgia from immune checkpoint inhibitor therapy is commonly reported as “low grade”, i.e., grade I or II, yet may develop long term chronicity in ~50% of patients who experience it. Grade II arthralgia is chronic pain that limits instrumental activities of daily living, and can have lasting patient impacts [4]. Integrating PROs is essential for enhancing patient-centered decision-making. This short communication advocates for several key practices to achieve such integration, including (1) fostering patient-centered communication by identifying factors contributing to discrepancies between physician- and patient-reported AEs in early-phase dose-finding trials, (2) facilitating partnerships among clinical trialists, statisticians, physicians, patients, and advocates to gain diverse perspectives of adverse events and in so doing ensure that patients comprehend how their data will be used, and (3) optimizing the integration of PROs in dose assignment decisions in early-phase trials.

## 2. Fostering Patient-Centered Communication

Growing evidence suggests that physician-reported AEs do not always align with a patient’s personal experience of tolerability. While physicians may easily assess objective AEs like fever, subjective AEs such as fatigue (QOL) often lack objective measurement, potentially leading to underreporting or misinterpretation of a patient’s actual burden of treatment [5]. These discrepancies in severity and frequency of patient-reported AEs compared to physician reports underscore the importance of clear, effective physician–patient communication about patient-reported outcomes (PROs) and their role in dose-finding trials. Prior research by our group [6], as well as by others [7], demonstrates that effective physician–patient communication is essential to foster high levels of patient understanding about clinical trials and involvement in trial decision-making. However, many physicians face challenges initiating discussions about clinical trials and struggle to reconcile their dual roles as caregivers and researchers [8]. These communication difficulties may contribute to patient misconceptions and knowledge gaps [9,10], such as overestimating the potential benefits of treatments [2,9,11,12,13,,14,15], undervaluing the importance of maintaining quality of life as the disease progresses [9], and having an incomplete understanding of their rights to abstain from or withdraw from a trial [16].

Communication challenges in Phase I trial settings differ somewhat from those in Phase II and III trials [17]. For instance, Phase I participants and are more influenced by factors such as their medical condition, their physician’s recommendation, hope for disease control, and a perceived lack of alternatives compared to Phase III participants [18,19,20,21]. However, research on patient–provider communication in Phase I trials remains limited. Our previous research, conducted with a small sample of oncologists specializing in Phase I trials, revealed that patients often felt overwhelmed by the volume of information provided, lacked clarity about the likelihood of personal benefit, and were constrained in their decision-making by persuasive treatment recommendations [6]. These early findings have been supported by later research [19,21,22]. In contrast, a separate study found that 92% of Phase I trial recommendations were delivered within a shared decision-making framework [23]. These findings underscore the complexity of patient–provider communication in Phase I trials and suggest that such complexity may extend beyond the areas described to discrepancies between physician-reported adverse events (AEs) and patient-reported experiences.

Phase I clinical trials, while demonstrating the potential for solid tumor responses in certain studies [1], also carry a substantial risk of severe side effects [2]. Advances in treatment regimens in National Cancer Institute (NCI) sponsored Clinical Therapy Evaluation Program (CTEP) Phase I trials have contributed to increased response rates without increased toxicity in patients with solid tumors. Response rates have doubled from 9.6% between 2001 and 2005 to 18.0% between 2013 and 2019 [24]. In spite of such advances in response rates, research indicates that patients often hold overly optimistic expectations about the effectiveness of experimental treatments [10]. This phenomenon, known as “therapeutic misconception”, has raised past ethical concerns regarding patient understanding of these trials [11,12,14,15] that continue in the current Phase 1 context [25,26,27]. In dose-escalation studies, patients may harbor hopes of being assigned to higher-dose arms, potentially exposing themselves to increased toxicity risks [13]. Our previous research suggests that the way physicians frame information about Phase I trials can emphasize potential efficacy, inadvertently encouraging enrollment [6]. Extending this idea, we propose that such communication may foster unrealistic expectations, leading some patients to underreport AE’s due to concerns of being removed from the Phase I study. Further investigation is needed to evaluate the influence of physician communication on a patient’s reporting behavior and its ethical implications.

## 3. Facilitating Partnerships

Phase I clinical trial designs are evolving as investigators increasingly incorporate therapeutic intent into dose escalation studies. Medicare covers routine trial costs in studies that explicitly incorporate therapeutic intent in their aims [28]. Another driving force behind these innovative trial designs is the growing recognition of the patients’ voice in the drug development process [8,29]. Patient input can be integrated through the inclusion of PROs. Various mechanisms for incorporating PROs in Phase I studies have been proposed. Most notable among these is the development of opportunities for patients and their caregivers to be involved in collaborative relationships with physicians, industry partners and other trial stakeholders early in the trial design to drive the selection of PROs that match patients’ concerns [30]. A study by Alger et al. [31] suggests that patients generally supported the incorporation of PROs within dose-finding trials but showed some apprehensiveness as to how PROs may reduce the size of the recommended dose (and potentially efficacious effect). Increasingly, an OBD has been recognized in non-cytotoxic therapy trials and pharmacodynamic studies as a meaningful endpoint [32]. In some cases, the OBD may exist independently of the MTD. Some participants expressed reluctance to accurately report the severity of their symptoms through patient-reported outcomes (PROs) due to concerns that doing so might lead to the discontinuation of their treatment [31]. Clearly defining the pursuit of the OBD as a primary objective could help empower patients to contribute more openly to PROs, knowing that their input will be valued in optimizing their treatment. Taken further, patient advocates suggest the patient voice should be integrated throughout the trial not only in the development phase [33]. Patient and stakeholders can pose questions, influence decisions and provide a perspective about PROs that would otherwise not be available [7,34]. The successful implementation of PROs will depend on patient support, making it essential to align the goals of the PRO measures with patients’ priorities in the trial. Engaging patients as co-investigators and collaborators in early-phase research protocols and seeking their input during trial design can help ensure that PRO tools are meaningful and patient-centered.

Cancer centers, specifically NCI-designated cancer centers, have a unique opportunity to address the underrepresentation of the patient perspective in Phase I trials by leveraging their offices of Community Outreach and Engagement (COE). COEs are charged with integrating communities’ voices (e.g., patients) in the cancer center’s clinical care and research. As such, experts in COE serve as a bridge between cancer centers and their members (e.g., physicians and clinical trialists) and the communities they serve. Community engagement frameworks inform approaches to ensure impact. One such framework, the Community-to-Bench model [35] consists of three tenants that, if operationalized correctly, may enhance patient engagement in early-phase trials. The three tenets of this model—community in-reach, data democratization, and flipped research—provide actionable steps, including intentional community input in research from conceptualization to dissemination and transparency and accessibility of data. In the context of Phase I trials, incorporating the Community-to-Bench model and patient voice could promote rapid translation of trial data, particularly as patients would inform the protocol, such as recruitment materials and wording for the instruction of reporting adverse events, and patient-reported adverse event data interpretation and sharing.

## 4. Optimizing Trial Design and Data Collection

To effectively integrate PROs into early-phase dose-finding trials, research must prioritize the feasibility of PRO collection and the development of trial designs that support incorporating PROs into dose-selection decision-making. While concerns have been raised about the patient burden of completing scheduled PRO-CTCAE questionnaires [36], studies have demonstrated the feasibility of administering the full set of 78 PRO-CTCAE items across multiple time points [37]. Feasibility could be further enhanced by using a subset of PRO-CTCAE items tailored to assess key toxicities, condensing the tool to a core list of symptoms that encourages patient adherence [37].

Several statistical approaches have been developed since 2020 to incorporate PROs into dose-finding trials, though these methods generally lack patient input during their design. Lee et al. introduced the PRO-continual reassessment method (PRO-CRM), which extends the continual reassessment method (CRM) by simultaneously considering physician- and patient-defined toxicity thresholds to identify the maximum tolerated dose (MTD) [38]. The first extension models physician and patient dose-limiting toxicities (DLTs) independently, while the other two integrate both outcomes into a joint model, either marginally or fully. The lower of the two dose recommendations is selected for subsequent cohorts and MTD determination. Wages et al. applied a Bayesian variant of PRO-CRM (PRO-CRMB) in a Phase I study evaluating adjuvant hypofractionated whole pelvis radiation therapy (WPRT) for endometrial cancer [39].

Andrillon et al. introduced two time-to-event (TITE) generalizations of PRO-CRM: TITE-PRO-CRM and TITE-CRM+PRO [33]. TITE-PRO-CRM incorporates pending physician- and patient-reported data during the trial using weighted likelihoods, while TITE-CRM+PRO relies solely on final PRO data to estimate the MTD. Alger et al. proposed the utility-PRO-CRM (U-PRO-CRM), which employs a utility-based framework to assign doses [31]. Unlike previous methods, U-PRO-CRM identifies the dose where physician and patient DLT rates align most closely with a pre-specified utility curve. Wages and Lin developed the isotonic PRO (PRO-ISO) design, which uses a beta-binomial model to estimate DLT probabilities based on physician and patient outcomes [40]. The design ensures dose–toxicity monotonicity using the pool adjacent violators algorithm (PAVA) and selects the lower dose recommended by both criteria. They later extended PRO-ISO to handle late-onset toxicities, resulting in the TITE-PRO-ISO design. Simulation studies evaluating the operating characteristics of PRO-based dose-finding designs indicate that these methods can correctly identify the MTD in a high percentage of simulated trials, using sample sizes consistent with traditional phase I trials, typically ranging from 15 to 40 patients. In the simulation studies of PRO-CRM and PRO-ISO, both methods were evaluated under the same set of seven dose–toxicity scenarios. When the sample size was 18, the percentage of correct selection (PCS) of the MTD ranged from 45 to 62 for PRO-CRM and from 43.6 to 62.8 for PRO-ISO. As the sample size increased to 40, the PCS improved, ranging from 68 to 76 for PRO-CRM and from 56.8 to 76.4 for PRO-ISO.

Most current methods determine the MTD by selecting the lower dose recommended by physician- and patient-reported outcomes, but they often fail to address discrepancies between estimated and target DLT rates. This uniform weighting of physician and patient DLTs can lead to overly conservative dose recommendations, potentially overlooking tolerable but more effective doses. Further research is needed to optimize the balance between these weights, which is also influenced by the quality of patient–provider communication. Moreover, no existing statistical methods explicitly differentiate between objective and subjective DLTs when integrating PROs. Exploring these distinctions could pave the way for novel, more effective strategies to incorporate PROs into dose-finding trials. Ongoing efforts to actively involve patients in the development of these intricate designs can help trialists better refine the balance between competing priorities. Finally, while the designs described above use binary endpoints to define clinician- and patient-assessed DLTs, relevant endpoints can also be collected as ordinal or continuous data, depending on the specific assessment tools and study objectives. Extending existing methods to accommodate these data types would be an important area for further research.

## 5. Conclusions

Integrating patient-reported outcomes (PROs) into early-phase dose-finding trials is pivotal for advancing patient-centered oncology research. By enhancing communication, fostering collaborative partnerships, and refining trial methodologies, the field can more effectively align clinical practices with the experiences and needs of patients. Future efforts should prioritize the development of robust statistical methods and innovative frameworks to address the complexities of incorporating PROs into dose-finding studies, thereby improving therapeutic development. At Virginia Commonwealth University (VCU) Massey Comprehensive Cancer Center, upcoming research will explore the application of these approaches in the catchment area, aiming to ensure that trial designs are both inclusive and reflective of diverse patient populations. These advancements hold significant promise for achieving more informed, equitable, and patient-focused outcomes in early-phase clinical trials.

## Data Availability

This is a short communication in which no new data was generated.
